# Antidiabetic Effect of *Cyclocarya paliurus* Leaves Depends on the Contents of Antihyperglycemic Flavonoids and Antihyperlipidemic Triterpenoids

**DOI:** 10.3390/molecules23051042

**Published:** 2018-04-29

**Authors:** Yang Liu, Yanni Cao, Shengzuo Fang, Tongli Wang, Zhiqi Yin, Xulan Shang, Wanxia Yang, Xiangxiang Fu

**Affiliations:** 1 College of Forestry, Nanjing Forestry University, Nanjing 210037, China; lyang_188@sina.com (Y.L.); 182cyn@sina.com (Y.C.); shangxulan@njfu.edu.cn (X.S.); yangwanxia@njfu.com.cn (W.Y.); xxfu@njfu.edu.cn (X.F.); 2 Co-Innovation Center for Sustainable Forestry in Southern China, Nanjing Forestry University, Nanjing 210037, China; 3 Department of Forest and Conservation Sciences, University of British Columbia, 3041- 2424 Main Mall, Vancouver, BC V6T 1Z4, Canada; tongli.wang@ubc.ca; 4 Department of Natural Medicinal Chemistry, China Pharmaceutical University, Nanjing 10009, China; chyzq2005@126.com

**Keywords:** *Cyclocarya paliurus*, diabetes, flavonoids, triterpenoids, geographical locations, extraction solvents

## Abstract

*Cyclocarya paliurus* has been used commonly to treat diabetes in China. However, the effective components and the effect of plant origin remain unclear. In this study, *C. paliurus* leaves with different chemical compositions were selected from five geographical locations, and their effects on streptozotocin (STZ)-induced diabetic mice were evaluated with both ethanol and aqueous extracts. Glucose levels, lipid levels, and biomarkers of liver and kidney function were measured. The principal components of both *C. paliurus* ethanol and aqueous extracts from different geographical locations differed quantitatively and qualitatively. Results showed that *C. paliurus* extracts with better antihyperglycemic effects were characterized by higher contents of total flavonoids, especially quercetin-3-*O*-glucuronide and kaempferol-3-*O*-glucuronide. Furthermore, significantly negative correlations were found between triterpenoids contents and lipid levels. These results revealed the potential antihyperglycemic capacity of *C. paliurus* flavonoids and the antihyperlipidemic effect of *C. paliurus* triterpenoids. Thus, we suggest that the composition of *C. paliurus* compounds might help to design therapeutic alternatives for the treatment of diabetes mellitus. However, geographic origins and the extraction solvents can also affect the effectiveness of the treatment as these factors influence the chemical compositions and thereby the biological activities.

## 1. Introduction

Diabetes mellitus (DM) is a serious chronic metabolic disease characterized by a high blood glucose level and changes in protein and lipid metabolism [1]. DM occurs worldwide and is one of the major causes of nontraumatic limb amputation, renal disease, and blindness, and the population of DM patients is increasing [2]. According to World Health Organization projections, more than 300 million people will be affected by DM in 2025 [3]. The current therapies for DM include oral anti-diabetic drugs, such as biguanides, sulfonylureas, thiazolidinediones, a-glucosidase inhibitors, dipeptidyl peptidase-4 inhibitors, etc., which are mainly used as monotherapy or in combination. However, these drugs present many undesired side effects, such as lactic acid intoxication and hypoglycemia, and ultimately cannot control the glycemic level [4]. Therefore, the search for safer and more effective phytochemical constituents from natural products for counteracting DM remains an active area. Nowadays, many antidiabetic components have been obtained from plants, including polysaccharides, flavonoids, terpenoids, saponins, unsaturated fatty acids, and alkaloids [5,6,7].

*Cyclocarya paliurus* (Batal) Iljinskaja belongs to the Juglandaceae family and is widely distributed in mountainous regions of sub-tropical China [8]. Leaves of this plant are traditionally used in China as an ingredient in nutraceutical tea or drug formulations for the treatment of hypertension, diabetes mellitus, and hyperliposis [9,10,11,12]. Previous studies have reported that *C. paliurus* leaves contain abundant bioactive components including polysaccharides, triterpenoids, flavonoids, and phenolic compounds [13,14,15]. Thus, chemical identification and the anti-diabetic effect of *C. paliurus* extracts have attracted the attention of many scholars, however the constituents responsible for anti-diabetic effect still remains controversial. For example, previous studies have revealed that the water extract of *C. paliurus* could reduce postprandial triglyceride level in hyperlipidemic mice, and predicted polysaccharides to be the active components [16,17]. Polysaccharides of *C. paliurus* are also found to exhibit a lipid-lowering effect on high-fat-diet-induced rats [18,19]. However, it is demonstrated that polysaccharides did not appear to be the active anti-diabetic constituent, based on the comparison of anti-diabetic effects of *C. paliurus* ethanol extract (without polysaccharides) and aqueous extract in streptozotocin (STZ)-induced diabetic rats [20]. In addition, several studies have shown that triterpenic acid-enriched *C. paliurus* fraction or extracts possess better antihyperlipidemic activities in mice fed with high-fat-diet [21,22,23]. Thus, further investigation of the anti-diabetic activities of *C. paliurus* extracts with detailed component information is required.

To fill the knowledge gap mentioned above, in this study, *C. paliurus* leaves with different chemical compositions were selected from five geographical locations and their effects on streptozotocin (STZ)-induced diabetic mice were evaluated with both ethanol and aqueous extracts. Principal components analysis (PCA) and canonical correspondence analysis (CCA) were used to analyze the chemical variability and its relationship with anti-diabetic activities. The potential antihyperglycemic capacity of *C. paliurus* flavonoids and the antihyperlipidemic effect of *C. paliurus* triterpenoids were revealed. Taken together, this study provides essential information supporting the use of *C. paliurus* extract as a natural medicine for diabetic patients.

## 2. Results and Discussion

### 2.1. Chemical Compositions

**Figure 1:**
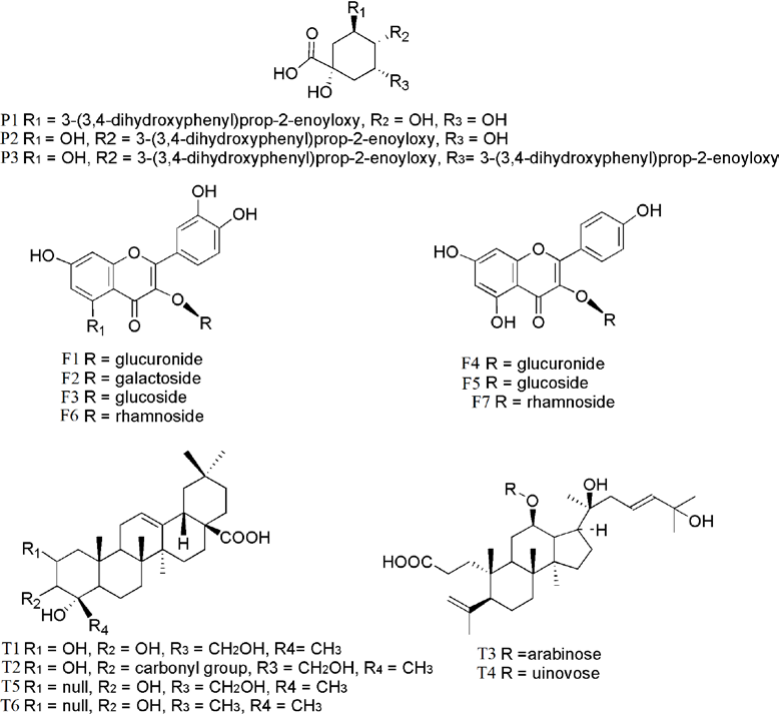
Chemical structures of identified compounds in extracts of *C. paliurus*: **P1**: 3-*O*-caffeoylquinic acid; **P2**: 4-*O*-caffeoylquinic acid; **P3**: 4,5-di-*O*-caffeoylquinic acid; **F1**: quercetin-3-*O*-glucuronide; **F2**: quercetin-3-*O*-galactoside; **F3**: isoquercitrin; **F4**: kaempferol-3-*O*-glucuronide; **F5**: kaempferol-3-*O*-glucoside; **F6**: quercetin-3-*O*-rhamnoside; **F7**: kaempferol-3-*O*-rhamnoside; **T1**: arjunolic acid; **T2**: cyclocaric acid B; **T3**: pterocaryoside B; **T4**: pterocaryoside A; **T5**: hederagenin; **T6**: oleanolic acid.

In order to have an idea about the compounds responsible for the anti-diabetic activities, phytochemical compositions of the extracts studied were clarified by using both qualitative and quantitative analyses. In total, 17 components, including polysaccharides, three phenolic acids, seven flavonoids, and six triterpenoids, were identified in ethanol and aqueous extracts of *C. paliurus* leaves from different geographical locations (Table 1; Figure 1). The major components were: 3-*O*-caffeoylquinic acid (**P1**, 0.04–1.38 mg/g), quercetin-3-*O*-glucuronide (**F1**, 0.30–2.38 mg/g), kaempferol-3-*O*-glucuronide (**F4**, 0.17–1.88 mg/g), kaempferol-3-*O*-rhamnoside (**F7**, 0.05–2.30 mg/g), arjunolic acid (**T1**, 0.02–2.70 mg/g), cyclocaric acid B (**T2**, 0.04–0.98 mg/g), pterocaryoside B (**T3**, 0.01–2.46 mg/g), and pterocaryoside A (**T4**, 1.07–2.48 mg/g). These results were comparable to those reported in our previous study [24]. The ethanol extracts from different locations were abundant in phenolic acids, flavonoids, and triterpenoids, but absent in polysaccharides. In contrast, aqueous extracts from different locations were observed abundant in polysaccharides, but absent in 4,5-di-*O*-Caffeoylquinic acid, pterocaryoside B, pterocaryoside A, and hederagenin. 

**Figure 2:**
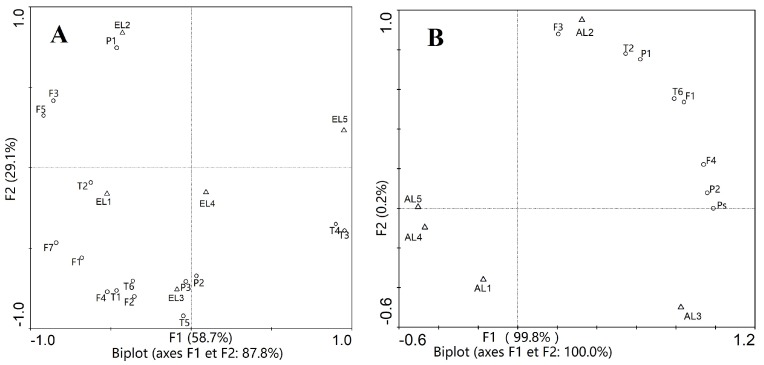
Biplots of Principal Component Analysis (PCA) of ethanol (**A**) and aqueous (**B**) extracts of *C. paliurus* from different locations. **L1**: Jinzhongshan; **L2**: Muchuan; **L3**: Wufeng; **L4**: Anji; **L5**: Suining. **P1**: 3-*O*-caffeoylquinic acid; **P2**: 4-*O*-caffeoylquinic acid; **P3**: 4,5-di-*O*-caffeoylquinic acid; **F1**: quercetin-3-*O*-glucuronide; **F2**: quercetin-3-*O*-galactoside; **F3**: isoquercitrin; **F4**: kaempferol-3-*O*-glucuronide; **F5**: kaempferol-3-*O*-glucoside; **F7**: kaempferol-3-*O*-rhamnoside; **T1**: arjunolic acid; **T2**: cyclocaric acid B; **T3**: pterocaryoside B; **T4**: pterocaryoside A; **T5**: hederagenin; **T6**: oleanolic acid; Ps: polysaccharides.

Results showed that chemical compositions of *C. paliurus* extracts varied significantly among the geographical locations (Table 1). The highest contents of total phenolic acids, total flavonoids, and total triterpenoids (1.57 mg/g, 6.88 mg/g, 8.15 mg/g) were observed from *C. paliurus* ethanol extract of Muchuan, Jinzhongshan, and Wufeng, respectively, while the lowest (0.06 mg/g, 0.52 mg/g, 0.05 mg/g) were all observed from *C. paliurus* aqueous extract of Anji. PCA results for the ethanol extracts indicated that the first principal component was corresponding to cyclocaric acid B (**EL1**), 3-*O*-caffeoylquinic acid (**EL2**), 4,5-di-*O*-caffeoylquinic acid, kaempferol-3-*O*-glucuronide, arjunolic acid, cyclocaric acid B, hederagenin, oleanolic acid (**EL3**), and pterocaryoside B, pterocaryoside A (**EL5**) (Figure 2A), while for the aqueous extracts, the first component was associated with isoquercitrin (**AL2**), and polysaccharides, 4-*O*-caffeoylquinic acid, kaempferol-3-*O*-glucuronide (**AL3**) (Figure 2B). PCA results in our study confirmed that geographic variation and extraction solvents influenced the bioactive components in both quantitative and qualitative extents [24,25].

### 2.2. Variation in Anti-Diabetic Properties

Streptozotocin (STZ) is a compound commonly used to induce diabetes in animal models, which could induce severe damage to the β-cells and then exhibit diabetes symptoms such as hyperglycemia and glucose intolerance [26]. In our experiment, the mice were injected intraperitoneally at a dose of 40 mg per kg per day STZ for five consecutive days (*n* = 6). Mice injected with STZ have been commonly used as models to study the antihyperglycemic and antihyperlipidemic effects of plant extracts on mammals, such as grape skin extract (*n* = 8), Boswellia serrata (*n* = 4), and peony seed oil (*n* = 7) [27,28,29]. In our study, the administration of *C. paliurus* extracts to STZ-induced diabetic mice was used to investigate the effects on various parameters, including body weight change, fasting blood glucose level (FBG), oral glucose tolerance test (OGTT), insulin tolerance test (ITT), lipid level, and liver and kidney function, compared with the diabetic control (DC) group, non-diabetic control (NC) group, and positive control (Metformin Hydrochloride Tablets, MHT; Xiaoke Pill, XKP) groups.

#### 2.2.1. Variation in Body Weight, FBG, OGTT, and ITT

**Figure 3:**
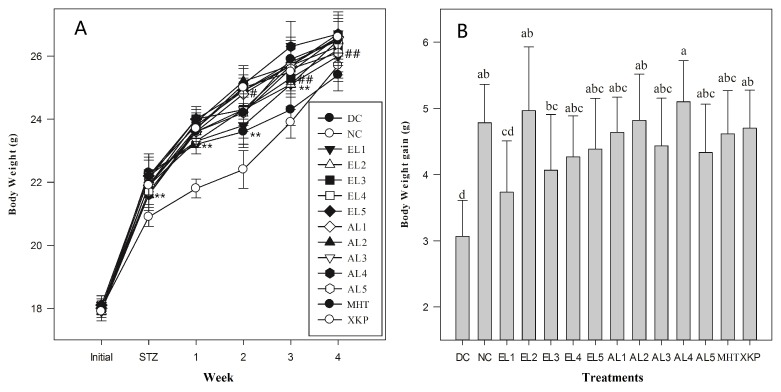
(**A**) Changes in body weight of different groups (*n* = 6). (**B**) Body weight gain. DC: diabetic control group; NC: normal control group; EL1-EL5: STZ-induced diabetic mice treated with ethanol extracts from different locations; AL1-AL5: STZ-induced diabetic mice treated with aqueous extracts from different locations; MHT, XKP: positive controls, STZ-induced diabetic mice treated with Metformin Hydrochloride Tablets (MHT) and Xiaoke Pill (XKP), respectively. Values with different letters significantly differ at *p* < 0.05 by Duncan’s test. # *p* < 0.05 and ## *p* < 0.01 significance against the normal control group (NC). **p < 0.01 significance against the model control group (DC).

**Figure 4:**
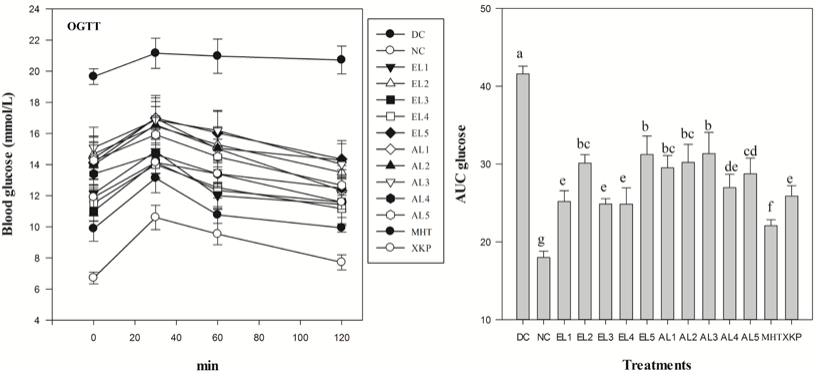
Levels of blood glucose in STZ-induced diabetic mice as revealed by oral glucose tolerance tests (OGTT) (*n* = 6). AUC glucose: area under the curve for glucose levels. DC: diabetic control group; NC: normal control group; EL1-EL5: STZ-induced diabetic mice treated with ethanol extracts from different locations; AL1-AL5: STZ-induced diabetic mice treated with aqueous extracts from different locations; MHT, XKP: positive controls, STZ-induced diabetic mice treated with Metformin Hydrochloride Tablets (MHT) and Xiaoke Pill (XKP), respectively. Values with different letters significantly differ at *p* < 0.05 by Duncan’s test.

**Figure 5:**
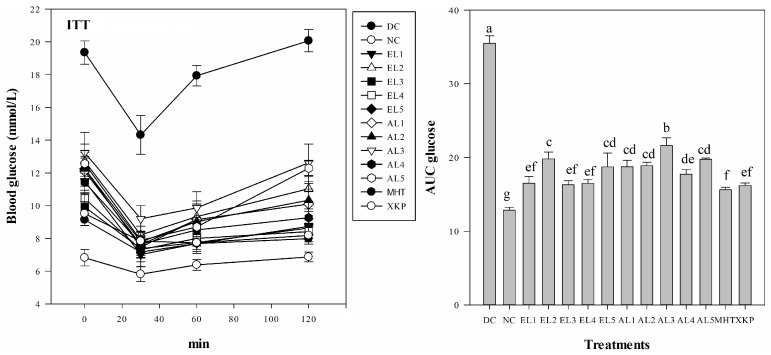
Levels of blood glucose in STZ-induced diabetic mice as revealed by insulin tolerance test (ITT) (*n* = 6). AUC glucose: area under the curve for glucose levels. DC: diabetic control group; NC: normal control group; EL1-EL5: STZ-induced diabetic mice treated with ethanol extracts from different locations; AL1-AL5: STZ-induced diabetic mice treated with aqueous extracts from different locations; MHT, XKP: positive controls, STZ-induced diabetic mice treated with Metformin Hydrochloride Tablets (MHT) and Xiaoke Pill (XKP), respectively. Values with different letters significantly differ at *p* < 0.05 by Duncan’s test.

Results suggested that both *C. paliurus* ethanol and aqueous extracts were beneficial to reverse body weight loss and to reduce glucose levels in FBG, OGTT, and ITT tests of STZ-induced diabetic mice, however it differed significantly among different groups (Figure 3, Figure 4 and Figure 5). After five days of STZ injection, the blood glucose level of FBG in all treated groups were significantly increased in comparison to those in the NC group (*p* < 0.05), which is consistent with previous studies on STZ-induced diabetic model for other plant extracts [29]. After administration with *C. paliurus* extracts or MHT, XKP for one week, a positive effect on FBG in STZ-induced diabetic mice were observed (Table 2). FBG final values of different treatments differed significantly (*p* < 0.05), and the lowest value of FBG was observed at EL3 treatment (9.8 mmol /L) (Table 2). The blood glucose level of OGTT in the DC group was significantly higher than that of the NC group (*p* < 0.01) (Figure 4), indicating that the glucose tolerance of the STZ-induced diabetic mice was severely impaired. The area under the curve (AUC) for OGTT of *C. paliurus* extracts differed and was significantly less in the presence of EL1 (25.1), EL3, and EL4 (24.9), which was comparable with that of XKP positive group (*p* < 0.05) (Figure 4). AUC of ITT (Figure 5) showed a similar pattern with OGTT, which indicated that EL1, EL3, and EL4 treatments had better antihyperglycemic effects on STZ-induced diabetic mice. 

**Figure 6:**
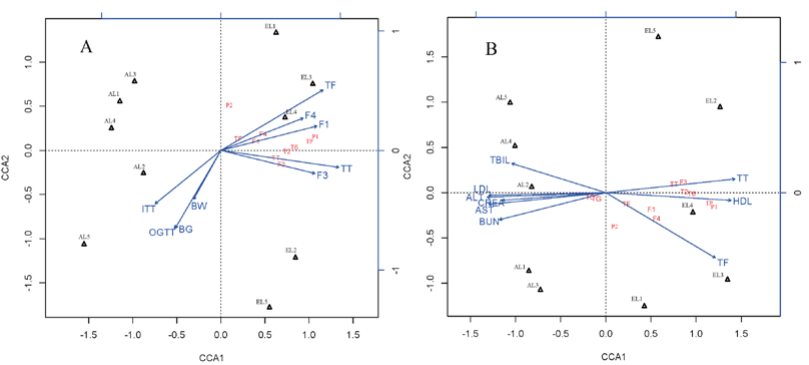
Biplots of Canonical Correspondence Analysis (CCA) of *C. paliurus* extracts. CCA were done based on matrix linking contents of the major components, geographical locations and their antihyperglycemic (**A**), antihyperlipidemic (**B**) activities in STZ-induced diabetic mice. BW, Body weight gain; BG, Final blood glucose level in fasting blood glucose test; OGTT, Oral glucose tolerance test; ITT, Insulin tolerance test; TG, Triglyceride; TC, Total cholesterol; LDL-c, Low density lipoprotein cholesterol; HDL-c, High-density lipoprotein cholesterol; BUN, Blood urea nitrogen; CREA, Creatinine; TBIL, Total bilirubin; AST, Aspartate aminotransferase; ALT, Alanine aminotransferase.

These results may be due to the higher contents of total flavonoids (TF), especially quercetin-3-*O*-glucuronide (F1), and kaempferol-3-*O*-glucuronide (F4) found in the ethanol extract at these locations, which was confirmed by the significantly negative correlations between flavonoids contents and glucose levels (FBG, OGTT, and ITT) in the CCA test (Figure 6A). To our best knowledge, our study is the first to reveal the potential antihyperglycemic effects of flavonoid glycosides of this species. Antihyperglycemic effects of kaempferol and quercetin from other plants have been reported. For example, it was reported that supplementation of kaempferol and quercetin isolated from *Euonymus alatus* (Celastraceae) could prevent the development of diabetes mellitus in experimental animals [30]. Their results proved that kaempferol and quercetin significantly improved insulin-stimulated glucose uptake in mature 3T3-L1 adipocytes, and they also served as partial agonists in a peroxisome proliferator-activated receptor gamma (PPAR-g) reporter gene assay [30]. Quercetin has also been reported to accelerate the function of insulin receptor and glucose transporter 4 (GLUT 4) which in turn lead to elevated glucose uptake [31]. Thus, further analysis of the antihyperglycemic effect of kaempferol or quercetin glycosides from *C. paliurus* is needed.

#### 2.2.2. Variation in Lipid Levels

The undesired elevation of the serum lipids observed in the DC mice of this study indicated that STZ could induce the lipid abnormalities. The protective effect of plant extracts on diabetes was related to their hypolipidemic effects [32]. The administration of EL1-EL5, AL1-AL5, and MHT, XKP reversed the level of lipids significantly (*p* < 0.05). The lowest values of total cholesterol (TC) and low-density lipoprotein cholesterol (LDL-c) for *C. paliurus* extracts were observed in the EL3 group, while the highest values were found in the AL4 group (Table 3). Our results confirmed that *C. paliurus* ethanol extracts with higher contents of total triterpenoids had a better effect on the lipid levels in STZ-induced diabetic mice (Table 3), which was in consistence with previous studies [21,22,23]. CCA results also indicated that *C. paliurus* ethanol extracts from L2, L3, and L4 with significantly lower lipid levels were characterized by total triterpenoids (TT), isoquercitrin (F3), cyclocaric acid B (T2), and oleanolic acid (T6) (Figure 6B). Thus, the cholesterol-lowering effect of *C. paliurus* extracts was possibly due to the ability of *C. paliurus* triterpenoids (cyclocaric acid B and oleanolic acid) to decrease the serum level of apolipoprotein B48 and the expression of tumour necrosis factor (TNF-a) [21,22,23]. Cyclocaric acid B, a unique constituent detected from *C. paliurus* leaves, has been reported to have the ability to decrease the apolipoprotein B48 oversecretion in Caco-2 cells [15,23]. In addition, oleanolic acid has been shown to increase the HDL-C level and decrease the atherogenic lipid level [33]. However, whether *C. paliurus* triterpenoids works alone or together with other compounds needs to be further studied in the hyperlipidaemic animal models.

#### 2.2.3. Variation in Liver and Kidney Function

Liver and kidney are the key organs in the body involved in almost all biochemical pathways related to nutrient supply, growth, energy provision, fighting diseases, reproduction, and regulating homeostasis [34,35]. Reliable biomarkers to evaluate the liver and kidney function were shown in Table 4, including the activities of aspartate aminotransferase (AST) and alanine aminotransferase (ALT), serum blood urea nitrogen (BUN), creatinine (CREA), and total bilirubin (TBIL). Liver and kidney were found to be necrotized in STZ-induced diabetic mice, and a significant increase in the activities of AST and ALT, and TBIL, CREA, and BUN were observed (Table 4). However, treatments with EL1-EL5, AL1-AL5, and MHT, XKP resulted in a significant decrease in the activities of AST and ALT, serum TBIL, CREA, and BUN compared to the DC group (*p* < 0.05). Values of these biomarkers differed significantly among *C. paliurus* extracts studied (*p* < 0.05). The lowest values of AST (35.1 U/L), ALT (94.5 U/L), and CREA (18.2 μmol/L) for *C. paliurus* extracts were observed in the EL3 group, while the lowest values of TBIL (1.02 μmol/L) and BUN (11.0 mmol/L) were found in the EL2 and EL4 group, respectively (Table 4).

The development of diabetes often leads to the leakage of the circulatory system, and high levels of AST, ALT, TBIL, CREA, and BUN were often observed in STZ-induced diabetic mice [36,37], which is in agreement with our results (Table 4). Results indicated that *C. paliurus* extracts improved the liver and kidney function by decreasing the serum AST, ALT, and TBIL, CREA, and BUN levels in the diabetic mice. Moreover, significantly positive correlations were found between the values of AST, ALT, CREA, BUN, and lipid index (TC, TG, and LDL-c) (Figure 6B), which is in agreement with other reports in hyperlipidemic mice [38,39]. Overall, these results suggested that *C. paliurus* extracts could offer protection to liver and kidney by effectively lowering the lipid level. These findings, together with evidence collected from previous reports suggest that the composition of *C. paliurus* compounds might help for the design of therapeutic alternatives for the treatment of diabetes mellitus. However, geographic origins and the extraction solvents can also affect the effectiveness of the treatment as these factors influence the chemical compositions and their antihyperglycemic and antihyperlipidemic activities.

## 3. Materials and Methods

### 3.1. Plant Materials 

Five geographical locations from five provinces in China for *C. paliurus* sampling were identified based on the major distribution of the species. Voucher specimens were deposited in Silviculture Lab of Nanjing Forestry University (Voucher code: 2011GX, 2011SC, 2011HB, 2011ZJ, 2011HN). The detailed geographic, climatic information, and soil index of the sample locations are listed in Table 5. Leaf samples were collected from each location in September 2014. At each location, 6–30 trees (generally dominant or co-dominant tree in the stand) were selected based on tree age (over 20 years-old), stem form, and growth vigor. Number of trees for collecting leaves for each location was determined according to quantity and stand area of *C. paliurus* which distributed on the area (about 10 % of the total). About 400 g fresh fully developed leaves were collected from the middle crown for each tree and then sealed up with silica gel for transportation. Leaves collected in each location were mixed together in the lab and then dried at 70 °C for 48 h to constant weight, and ground into fine powder before extraction. All samples were stored at room temperature prior to analysis.

### 3.2. Chemical Reagents and References 

Streptozotocin (STZ) was purchased from Sigma Chemical Co. (St. Louis, MO, USA). Metformin Hydrochloride Tablets (MHT) was bought from Sino-American Shanghai Squibb Pharma (Shanghai, China), and Xiaoke Pill (XKP) from Guangzhou Zhongyi Pharmaceutical Enterprise (Guangdong, China). Commercial test kits used for measuring TG, TC, LDL-c, HDL-c, BUN, CREA, TBIL, AST, and ALT were purchased from Jiancheng Institute of Biotechnology (Nanjing, China). The reference standards of 3-*O*-caffeoylquinic acid, 4-*O*-caffeoylquinic acid, isoquercitrin, 4,5-di-*O*-caffeoylquinic acid, kaempferol-3-*O*-glucuronide, quercetin-3-*O*-glucuronide, arjunolic acid, quercetin-3-*O*-rhamnoside, quercetin-3-*O*-galactoside, and kaempferol-3-*O*-glucoside (purity >98%) were purchased from BioBioPha Co., Ltd. (Kunming, China), and arjunolic acid, oleanolic acid (purity >98%) were purchased from Shanghai Yuanye Biotechnology Co., Ltd. (Shanghai, China), whereas cyclocaric acid B, kaempferol-3-*O*-rhamnoside, and pterocaryoside A and pterocaryoside B (purity >98%) were isolated and purified from the laboratory of China Pharmaceutical University (Nanjing, China) [24]. 

### 3.3. Preparation of Ethanol and Aqueous Extracts 

The leaf powder (about 75g) from each location was extracted with 80% ethanol (1 L) and distilled water (1 L), respectively, and then incubated in a water bath at 90 °C for 2 h. Bottles containing samples were shaken on a shaker at room temperature for 15 min. After centrifugation at 8000 g for 15 min, the supernatant was transferred into a new bottle. Supernatants were then concentrated at 40 °C to yield an 80% ethanol or aqueous extract. The samples (**L1**–**L5**) extracted with 80% ethanol were named **EL1** (36.5g, 48.7% yield), **EL2** (37.6g, 50.1% yield), **EL3** (36.8g, 49.1% yield), **EL4** (36.1g, 48.1% yield), and **EL5** (36.4g, 48.5% yield), while the samples (**L1**–**L5**) extracted with distilled water were named **AL1** (42.1g, 56.1% yield), **AL2** (39.8g, 53.1% yield), **AL3** (40.9g, 54.5% yield), **AL4** (43.5g, 58.0% yield), and **AL5** (40.2g, 53.6% yield), respectively. All extracts were stored at 4 °C before chemical analysis and animal test.

### 3.4. Determination of Chemical Compositions 

Polysaccharide contents of the extracts were determined by the phenol-sulfuric acid method using d-glucose as a standard [40]. Phenolic acids, flavonoids, and triterpenoids profiles of the extracts were analyzed using an HPLC system, as described in our previous study [24]. Briefly, the extracts were filtered through a 0.22 μm polytetrafluoroethylene (PTFE) filter, then analyzed using a Waters e2695 Alliance (Waters Corp., Milford, MA, USA) equipped with Waters 2695 separation unit (an auto sampler, an online degasser, a quaternary pump solvent management system, a gasket cleaning system and a column heater), an Empower 3 data processing system and a Waters 2489 ultraviolet detector (UVD). Chromatographic separation was carried out on an X-Bridge C18 column of 250 × 4.6 mm packed with 5 μm particles by a stepwise elution with water containing 0.01% (*v*/*v*) formic acid (solution A) and acetonitrile containing 0.01% (*v*/*v*) formic acid (solution B). The gradient program was as follows: 0–13 min ratio of solution A to solution B 92:8 (*v*/*v*), 13-28 min 81:19, 28-42 min 79:21, 42–60 min 50:50, 60–64 min 55:45, 64–74 min 44:56, 74–90 min 34:66, 90–95 min 15:85, and 95–100 min 0:100. The flow rate was 1.0 mL/min, the injection volume was 10 μL, and the column temperature was 45 °C. The wavelength of monitor was set at 205 nm. Contents of individual phenolic acids, flavonoids, and triterpenoids were quantified on the basis of their external standards. Total phenolic acids (TP), total flavonoids (TF) and total triterpenoids (TT) were the sum of their individuals, respectively.

### 3.5. Animals and Experimental Design 

C57BL/6 mice (male, weighing 17.9–18.1 g, certificate No. SCXK (HU) 2013-0018) were purchased from Shanghai Lingchang Biotechnology Corporation and housed under controlled temperature (22 ± 2 °C) and humidity (55% ± 5%). Animals were supplied with water and food in accordance with guidelines and policy set forth by the Chinese Experimental Animals Administration Legislation (Ethical Protocol# SL-008-01). Mice of non-diabetic control (NC) were fed with a basal control diet. While for the development of diabetes, mice were fed with high fat diet (HFD), consisting of 20% carbohydrate, 3% egg, 18% fat, and 59% basic diet (*w/w*) [41]. Six weeks later, mice fed with high fat diet (HFD) were injected intraperitoneally with a dose of 40 mg/kg streptozotocin (STZ) incitrate buffer (pH = 4.5) for five consecutive days. The blood samples were collected and mice with blood glucose > 14 mmol/L were used for anti-diabetic study. The samples were tested for anti-diabetic activity at a concentration of 8 g/kg/day with a basal control diet for four weeks, based on our previous study [20]. MHT and XKP were used as positive controls, while distilled water was used as a negative one. 

Animals were divided in 14 groups of 6 mice each as follow:

Group 1: diabetic control (DC), STZ-induced diabetic mice treated with 10 mL distilled water per kg body weight once a day.

Group 2: non-diabetic control (NC), normal mice treated with 10 mL distilled water per kg body weight once a day.

Group 3, 4, 5, 6, 7: STZ-induced diabetic mice treated with 8 g per kg body weight of EL1–EL5 once a day, respectively.

Group 8, 9, 10, 11, 12: STZ-induced diabetic mice treated with 8 g per kg body weight of AL1–AL5 once a day, respectively.

Group 13: STZ-induced diabetic mice treated with 250 mg per kg body weight of Metformin Hydrochloride Tablets (MHT) once a day.

Group 14: STZ-induced diabetic mice treated with 1.73 g per kg body weight of Xiaoke Pill (XKP) once a day [20].

### 3.6. Body Weight and Fasting Blood Glucose Level (FBG) 

Body weight and FBG level of all the animal groups were determined weekly after treatment. Body weight gain was calculated from the equation: Body weight gain = BW_28day_–BW_0day_, where BW_28day_ is value of body weight after 28-days’ treatment and BW_0day_ is the initial body weight after STZ injected. Tail vein blood was collected weekly and concentrations of glucose were determined with glucose kits. 

### 3.7. Oral Glucose Tolerance Test (OGTT) and Insulin Tolerance Test (ITT) 

OGTT of each group was performed on the 28th day of the treatment, according to the reported method [20]. Animals of each group were administered glucose (2.5 g/kg, i.g.) before test. The blood samples were collected from the orbital venous plexus just before glucose load (0 min) and at 30, 60, and 120 min after glucose administration. ITT of each group was performed on the third day after OGTT, according to the previous method [20]. Animals of each group were administered insulin (1.0 U/kg, subcutaneous injection) before test. The blood samples were collected from the orbital venous plexus just before insulin administration (0 min), and at 30, 60, and 120 min after insulin administration. AUC of glucose concentrations over time was calculated by the trapezoidal rule.

### 3.8. Biochemical Analyses of the Serum Samples 

The blood samples were collected from abdominal aortic at the end of animal trial and centrifuged immediately for 5 min at 12,000 g at 4 °C to obtain serum for biochemical analysis. TG, TC, LDL-c, and HDL-c were measured using commercially available kits according to the manufacturer's directions.

### 3.9. Liver and Kidney Function 

Upon the end of the animal trial, indexes of liver and kidney function were measured at the same time of biochemical analyses of the serum samples. BUN, TBIL, AST, and ALT were measured using commercially available kits according to the manufacturer's directions.

### 3.10. Statistical Analysis 

Chemical variability and anti-diabetic activities of *C. paliurus* extracts among different treatments were compared with an analysis of variance (ANOVA, GLM procedure, *p* < 0.05) and Duncan multiple range tests, using SPSS software version 16.0 (SPSS, Chicago, IL, USA). To assess the chemical variability of extracts from different locations, we used PCA based on matrix linking contents of the major components from ethanol and aqueous extracts to different locations. The multivariate analysis was carried out using SPSS software (SPSS, Chicago, IL, USA). The impact of chemical variability on the antihyperglycemic and antihyperlipidemic properties in STZ-induced mouse model was assessed over CCA, using multi-variate statistical package (MVSP) V5.2. 

## 4. Conclusions

In conclusion, based on the analysis of chemical variability and its relationship with anti-diabetic activities, our results revealed the potential antihyperglycemic capacity of *C. paliurus* flavonoids and the antihyperlipidemic effect of *C. paliurus* triterpenoids, while these potential capacities were closely related to their contents. Further studies on the antihyperglycemic effect of *C. paliurus* kaempferol or quercetin glycosides and the antihyperlipidemic mechanism of *C. paliurus* triterpenoids will be carried out in future research.

## Figures and Tables

**Table 1:** Chemical compositions of ethanol and aqueous extracts in *C. paliurus* from different geographical locations (mg/g, mean ± SD). Different letters indicate significant differences (*p* < 0.05 by Duncan’s test) between treatments (*n* = 3) (nd, not detected).

Compounds	LOD (ng/mL)	LOQ (ng/mL)	EL1	EL2	EL3	EL4	EL5	AL1	AL2	AL3	AL4	AL5
3-O-caffeoylquinic acid (**P1**)	64.25	214.21	0.35 ± 0.011 cd	1.38 ± 0.153 a	0.39 ± 0.003 cd	0.45 ± 0.028 bc	0.31 ± 0.003 d	0.09 ± 0.003 e	1.30 ± 0.028 a	0.55 ± 0.006 b	0.04 ± 0.003 e	0.09 ± 0.006 e
4-O-caffeoylquinic acid (**P2**)	57.93	197.58	0.03 ± 0.001 h	0.08 ± 0.003 e	0.28 ± 0.003 b	0.11 ± 0.003 d	0.08 ± 0.002 e	0.04 ± 0.001 g	0.23 ± 0.003 c	0.35 ± 0.003 a	0.01 ± 0.001 i	0.05 ± 0.003 f
4,5-di-O-caffeoylquinic acid (**P3**)	58.97	201.22	0.05 ± 0.003 d	0.10 ± 0.001 c	0.42 ± 0.028 a	0.16 ± 0.003 b	0.08 ± 0.003 cd	nd	nd	nd	nd	nd
quercetin-3-*O*-glucuronide (**F1**)	40.28	128.74	2.15 ± 0.010 b	1.57 ± 0.006 d	2.12 ± 0.003 c	2.38 ± 0.005 a	0.78 ± 0.003 g	0.33 ± 0.003 h	1.25 ± 0.028 e	0.98 ± 0.003 f	0.30 ± 0.009 i	0.34 ± 0.009 h
quercetin-3-*O*-galactoside (**F2**)	52.94	174.17	0.47 ± 0.005 c	0.24 ± 0.003 d	0.52 ± 0.003 b	0.57 ± 0.005 a	0.20 ± 0.003 e	0.02 ± 0.001 g	0.01 ± 0.001 h	nd	nd	0.05 ± 0.001 f
isoquercitrin (**F3**)	58.42	192.52	0.22 ± 0.005 b	0.32 ± 0.006 a	0.18 ± 0.006 c	0.21 ± 0.013 b	0.08 ± 0.001 d	0.01± 0.001 g	0.05± 0.006 e	0.02 ± 0.001 fg	0.02 ± 0.003 fg	0.03 ± 0.001 ef
kaempferol-3-O-glucuronide (**F4**)	43.98	153.14	1.33 ± 0.001 b	0.86 ± 0.001 d	1.88 ± 0.001 a	0.95 ± 0.001 c	0.51 ± 0.001 g	0.18 ± 0.001 i	0.61 ± 0.001 f	0.74 ± 0.001 e	0.20 ± 0.001 h	0.17 ± 0.001 i
kaempferol-3-O-glucoside (**F5**) glucoside (**F5**)	53.85	187.37	0.18± 0.001 b	0.20 ± 0.001 a	0.12 ± 0.001 c	0.08 ± 0.001 d	0.05 ± 0.001 e	nd	0.02 ± 0.002 f	nd	nd	nd
quercetin-3-O-rhamnoside (**F6**) rhamnosiderhamnoside (F6)	62.48	199.32	0.23 ± 0.001 a	0.11 ± 0.001 d	0.20 ± 0.001 b	0.19 ± 0.001 c	nd	0.01 ± 0.001 g	0.01 ± 0.001 f	0.04 ± 0.001 e	nd	nd
kaempferol-3-O-rhamnoside (**F7**)	64.13	211.81	2.30 ± 0.001 a	1.39 ± 0.001 d	1.78 ± 0.001 b	1.50 ± 0.006 c	0.68 ± 0.001 e	nd	0.05 ± 0.001 g	0.25 ± 0.001 f	nd	nd
arjunolic acid (**T1**)	78.13	260.17	2.25 ± 0.001 b	1.65 ± 0.0011 c	2.70 ± 0.001 a	1.60 ± 0.001 d	1.47 ± 0.001 e	0.02 ± 0.001 g	0.07 ± 0.001 f	0.07 ± 0.001 f	nd	0.02 ± 0.001 g
cyclocaric acid B (**T2**)	90.16	296.36	0.71 ± 0.001 c	0.95 ± 0.001 b	0.98 ± 0.001 a	0.65 ± 0.001 d	0.55 ± 0.001 e	0.05± 0.004g h	0.07 ± 0.001 f	0.06 ± 0.001 g	0.04 ± 0.002 h	0.05 ± 0.001 gh
pterocaryoside B (**T3**)	72.46	210.27	0.24 ± 0.001 d	0.01 ± 0.001 e	1.61 ± 0.008 b	1.42 ± 0.002 c	2.46 ± 0.002 a	nd	nd	nd	nd	nd
pterocaryoside A (**T4**)	86.39	268.62	1.66 ± 0.007 d	1.07 ± 0.004 e	1.73 ± 0.003 c	2.03 ± 0.002 b	2.48 ± 0.006 a	nd	nd	nd	nd	nd
hederagenin (**T5**)	72.82	232.30	0.56 ± 0.002 c	0.45 ± 0.002 e	0.81 ± 0.004 a	0.70 ± 0.001 b	0.52 ± 0.003 d	nd	nd	nd	nd	nd
oleanolic acid (**T6**)	58.41	185.72	0.26 ± 0.002 c	0.25 ± 0.001 c	0.31 ± 0.001 a	0.30 ± 0.002 b	0.22 ± 0.002 d	0.003 ± 0.0001 fg	0.009 ± 0.0001 e	0.007 ± 0.0002 ef	0.001 ± 0.0001 g	0.003 ± 0.0001 fg
total phenolic acids (TP)	-	-	0.43 ± 0.014 e	1.57 ± 0.154 a	1.09 ± 0.023 b	0.71 ± 0.034 d	0.47 ± 0.007 e	0.12 ± 0.003 f	1.53 ± 0.026 a	0.91 ± 0.003 c	0.06 ± 0.003 f	0.13 ± 0.009 f
total flavonoids (TF)	-	-	6.88 ± 0.011 a	4.70 ± 0.002 d	6.80 ± 0.006 b	5.90 ± 0.008 c	2.30 ± 0.001 e	0.55 ± 0.003 i	1.99 ± 0.021 g	2.03 ± 0.001 f	0.52± 0.003 j	0.60 ± 0.010 h
total triterpenoids (TT)	-	-	5.69 ± 0.012 d	4.39 ± 0.004 e	8.15 ± 0.018 a	6.70 ± 0.008 c	7.70 ± 0.001 b	0.08 ± 0.004 g	0.15 ± 0.002 f	0.13 ± 0.001 f	0.05 ± 0.003 h	0.07 ± 0.001 g
polysaccharides (Ps)	-	-	nd	nd	nd	nd	nd	34.5 ± 2.02 c	44.0 ± 5.54 b	53.6 ± 4.05 a	28.8 ± 4.91 cd	28.2 ± 3.07 d

**Table 2:** Levels of fasting blood glucose (FBG) in experimental mice of different groups over the 28-day trial (mean ± SD). Different letters indicate significant differences (*p* < 0.05 by Duncan’s test) between treatments (*n* = 6).

Treatment	Fasting blood glucose level (mmol /L)				
	0 day	7 day	14 day	21 day	28 day
DC	17.9 ± 0.54 a	19.0 ± 0.46 a	19.2 ± 0.75 a	19.4 ± 0.82 a	20.0 ± 0.73 a
NC	6.6 ± 0.31 b	6.2 ± 0.27 e	6.3 ± 0.27 g	6.1 ± 0.44 g	6.1 ± 0.28 g
EL1	16.8 ± 0.43 a	15.8 ± 0.69 cd	14.9 ± 0.73 bcde	12.0 ± 0.87 cde	10.6 ± 0.48 de
EL2	16.8 ± 0.88 a	15.5 ± 0.35 d	14.2 ± 0.51 cdef	14.3 ± 0.58 b	12.8 ± 0.89 b
EL3	17.8 ± 0.40 a	15.5 ± 0.42 d	13.3 ± 0.95 ef	10.7 ± 0.61 ef	9.8 ± 0.45 ef
EL4	16.8 ± 1.10 a	15.5 ± 0.82 d	13.2 ± 0.93 ef	10.9 ± 1.03 def	10.4 ± 1.20 e
EL5	17.9 ± 1.02 a	15.9 ± 0.71 cd	14.1 ± 0.78 def	13.2 ± 1.20 bc	12.4 ± 1.06 b
AL1	17.4 ± 1.01 a	16.6 ± 0.55b cd	15.0 ± 1.10b cde	13.7 ± 0.98 bc	11.8 ± 1.10 bcd
AL2	17.5 ± 0.78 a	16.3 ± 0.56b cd	15.2 ± 0.87b cde	12.7 ± 1.07 bcd	11.8 ± 1.49 bcd
AL3	18.1 ± 0.60 a	17.7 ± 1.63 ab	15.6 ± 1.30 bcd	13.5 ± 1.27 bc	12.8 ± 1.35 b
AL4	17.4 ± 1.22 a	18.4 ± 1.27 a	16.5 ± 1.16 b	12.4 ± 1.28 bcde	10.8 ± 1.34 cde
AL5	17.1 ± 0.78 a	17.3 ± 1.20 abc	16.2 ± 0.70 bc	13.1 ± 1.28 bc	12.1 ± 1.42 bc
MHT	17.4 ± 0.82 a	15.0 ± 0.85 d	12.6 ± 1.16 f	9.2 ± 0.74 f	8.5 ± 0.66 f
XKP	17.8 ± 0.57 a	15.7 ± 1.06 cd	13.3 ± 1.74 ef	10.8 ± 0.78 def	9.5 ± 0.59 ef

**Table 3:** Levels of serum lipids in different groups of experimental mice at the end of the diabetic trial (mean ± SD). Different letters indicate significant differences (*p* < 0.05 by Duncan’s test) between treatments (*n* = 6).

Treatment	TC (mmol/L)	TG (mmol/L)	HDL-c (mmol/L)	LDL-c (mmol/L)
DC	3.76 ± 0.19 a	3.09 ± 0.49 a	1.57 ± 0.25 c	0.31 ± 0.05 a
NC	2.64 ± 0.23 bcd	1.48 ± 0.30 c	1.87 ± 0.24 abc	0.20 ± 0.04 cd
EL1	2.39 ± 0.25 cd	1.21 ± 0.21 c	1.95 ± 0.24 abc	0.24 ± 0.04 abcd
EL2	2.08 ± 0.38 d	1.19 ± 0.23 c	2.11 ± 0.21 abc	0.21 ± 0.03 bcd
EL3	2.08 ± 0.15 d	1.15 ± 0.24 c	2.34 ± 0.39 a	0.18 ± 0.02 d
EL4	2.38 ± 0.21 cd	1.02 ± 0.13 c	2.23 ± 0.34 ab	0.23 ± 0.03 abcd
EL5	2.57 ± 0.49 bcd	1.21 ± 0.04 c	2.05 ± 0.31 abc	0.24 ± 0.04 abcd
AL1	2.66 ± 0.42 bcd	1.45 ± 0.37 c	1.66 ± 0.11 c	0.28 ± 0.03 abc
AL2	2.97 ± 0.32 bc	1.41 ± 0.29 c	1.78 ± 0.18 bc	0.28 ± 0.04 abc
AL3	3.03± 0.12 b	1.56 ± 0.16 c	1.74 ± 0.21 bc	0.29 ± 0.05 ab
AL4	3.10 ± 0.42 b	1.24 ± 0.12 c	1.58 ± 0.16 c	0.31 ± 0.04 a
AL5	2.95 ± 0.25 bc	1.29 ± 0.28 c	1.59 ± 0.08 c	0.25 ± 0.04 abcd
MHT	3.00 ± 0.19 b	2.35± 0.29 b	1.70 ± 0.24 bc	0.23 ± 0.02 abcd
XKP	3.12 ± 0.23 b	2.36 ± 0.34 b	1.93 ± 0.18 abc	0.27 ± 0.03 abcd

**Table 4:** Index of liver and kidney function in different groups of experimental mice at the end of the diabetic trial (mean ± SD). Different letters indicate significant differences (*p* < 0.05 by Duncan’s test) between treatments (*n* = 6).

Treatment	ALT (U/L)	AST (U/L)	TBIL (μmol/L)	CREA (μmol/L)	BUN (mmol/L)
DC	52.8± 2.17 a	145.6 ± 3.08 a	7.50 ± 0.89 a	25.8 ± 1.17 a	17.3 ± 0.99 a
NC	28.0 ± 1.84 e	99.8 ± 6.87 fg	1.00 ± 0.18 g	14.7 ± 1.36 e	9.80 ± 0.18 d
EL1	36.1 ± 2.63 cd	113.3 ± 8.10 def	1.20 ± 0.15 fg	20.8 ± 0.75 bcd	13.4 ± 0.86 b
EL2	35.9 ± 1.80 cd	94.8 ± 8.59 g	1.02 ± 0.02 g	19.2 ± 1.94 cd	12.6± 1.08 bc
EL3	35.1 ± 3.36 d	94.5 ± 4.45 g	1.19 ± 0.18 fg	18.2 ± 2.13 d	13.1 ± 1.26 bc
EL4	36.9 ± 3.35 bcd	95.8 ± 9.05 g	1.46 ± 0.16 fg	21.0 ± 0.89 bcd	11.0 ± 1.17 cd
EL5	36.2 ± 3.38 cd	114.0 ± 7.66 de	1.38 ± 0.18 fg	21.0 ± 1.26 bcd	12.7 ± 0.26 bc
AL1	40.3 ± 4.51 bcd	130.2 ± 4.56 b	1.47 ± 0.18 fg	21.7 ± 1.86 bc	14.7 ± 0.81 b
AL2	43.4 ± 6.36 b	128.9 ± 5.30 bc	2.03 ± 0.10 ef	22.3 ± 1.21 b	14.2 ± 0.69 b
AL3	41.6 ± 1.36 bcd	123.6 ± 6.69 bcd	2.57 ± 0.28 e	22.8 ± 0.98 b	14.3 ± 0.92 b
AL4	43.1± 5.16 b	125.8 ± 4.16 bcd	4.87 ± 0.17 bc	21.8 ± 1.17 bc	14.2 ± 1.17 b
AL5	40.0 ± 3.19 bcd	115.5 ± 6.08 cde	3.63 ± 0.21 d	21.2 ± 0.98 bc	14.2 ± 1.32 b
MHT	42.4 ± 2.06 bc	121.1± 6.06 bcde	5.60 ± 0.55 b	21.0 ± 1.67 bcd	14.3 ± 0.76 b
XKP	43.5 ± 2.39 b	108.2 ± 8.57 efg	4.41 ± 0.22 cd	22.0 ± 1.79 bc	13.9 ± 0.82 b

**Table 5:** Geographical, climatic information and soil index of locations for *C. paliurus* sampling. T: Annual mean temperature; SH: Annual mean sunshine hours; P: Annual mean Precipitation; Soil C: organic matter content in soil; Soil N: total nitrogen content in soil.

Sample code	Location	Latitude (N)	Longitude (E)	T (℃)	Altitude (m)	SH (h)	P (mm)	Soil index						
								pH	C (%)	N (%)	P (%)	K (%)	Ca (%)	Mg (%)
**L1**	Jinzhongshan	24°36′36″	104°57′00″	17.1	1798	1475	1200.0	3.97	5.15	0.44	0.35	0.55	0.06	0.33
**L2**	Muchuan	28°58′00″	103°47′00″	12.9	1200	965.3	1533.2	3.81	3.58	0.27	0.08	0.98	0.19	0.22
**L3**	Wufeng	30°17'00″	110°80'00″	16.7	688	1533	1893.9	4.38	4.18	0.27	0.12	1.44	0.39	0.41
**L4**	Meiwu	27°46'00″	119°17'00″	16.5	678	1862	1600.0	4.48	6.46	0.45	0.22	2.03	0.18	0.22
**L5**	Suining	26°22'24″	110°07'47″	16.7	862	1348	1320.0	4.61	3.17	0.38	0.13	1.28	0.22	0.22
